# Diprovocim protects against the radiation-induced damage via the TLR2 signaling pathway

**DOI:** 10.1186/s10020-025-01198-2

**Published:** 2025-04-17

**Authors:** Duo Fang, Hainan Zhao, Lu Pei, Kai Jiang, Yuhan Gan, Xuanlu Zhai, Liao Zhang, Ying Cheng, Cong Liu, Jicong Du, Fu Gao

**Affiliations:** 1 School of Health Science and Engineering, University of Shanghai for Science and Technology, Shanghai, 200093 People’s Republic of China; 2 Department of Radiation Medicine, Faculty of Naval Medicine, Naval Medical University, 800 Xiangyin Road, Shanghai, 200433 People’s Republic of China; 3 Department of Radiology Intervention, Changhai Hospital Affiliated to the Naval Medical University, Shanghai, China; 4 Inner Mongolia Key Laboratory of Hypoxic Translational Medicine, Baotou Medical College, Baotou, 014040 China

**Keywords:** Ionizing radiation-induced injury, Intestinal stem cells, Diprovocim, TLR2, SOX9

## Abstract

Severe ionizing radiation (IR) causes the acute lethal damage of hematopoietic system and gastrointestinal tract. By establishing a radiation injury model, we found that Diprovocim, a TLR2 agonist, protected mice against the lethal damage of hematopoietic system and gastrointestinal tract. Diprovocim inhibited the IR-induced damage, promoted erythrocyte differentiation and elevated the proportion of hematopoietic stem cells (HSCs) in irradiated mice, and promoted the proliferation and differentiation of intestinal stem cells (ISCs). In addition, the RNA seq results suggested that Diprovocim significantly upregulated the TLR2 signaling pathway, and Diprovocim had no radioprotective effect on TLR2 KO mice, suggesting that Diprovocim activated TLR2 signaling pathway to exert its radioprotective function. The RNA sequencing results also suggested that Diprovocim significantly up-regulated the expression of SOX9. Diprovocim had no radioprotective effect after SOX9 knockdown. In conclusion, we demonstrated that Diprovocim protected the radiation-induced damage and upregulated targeting TLR2-SOX9 axis and that Diprovocim might be a potential high-efficiency selective agent.

**Graphical abstract:**

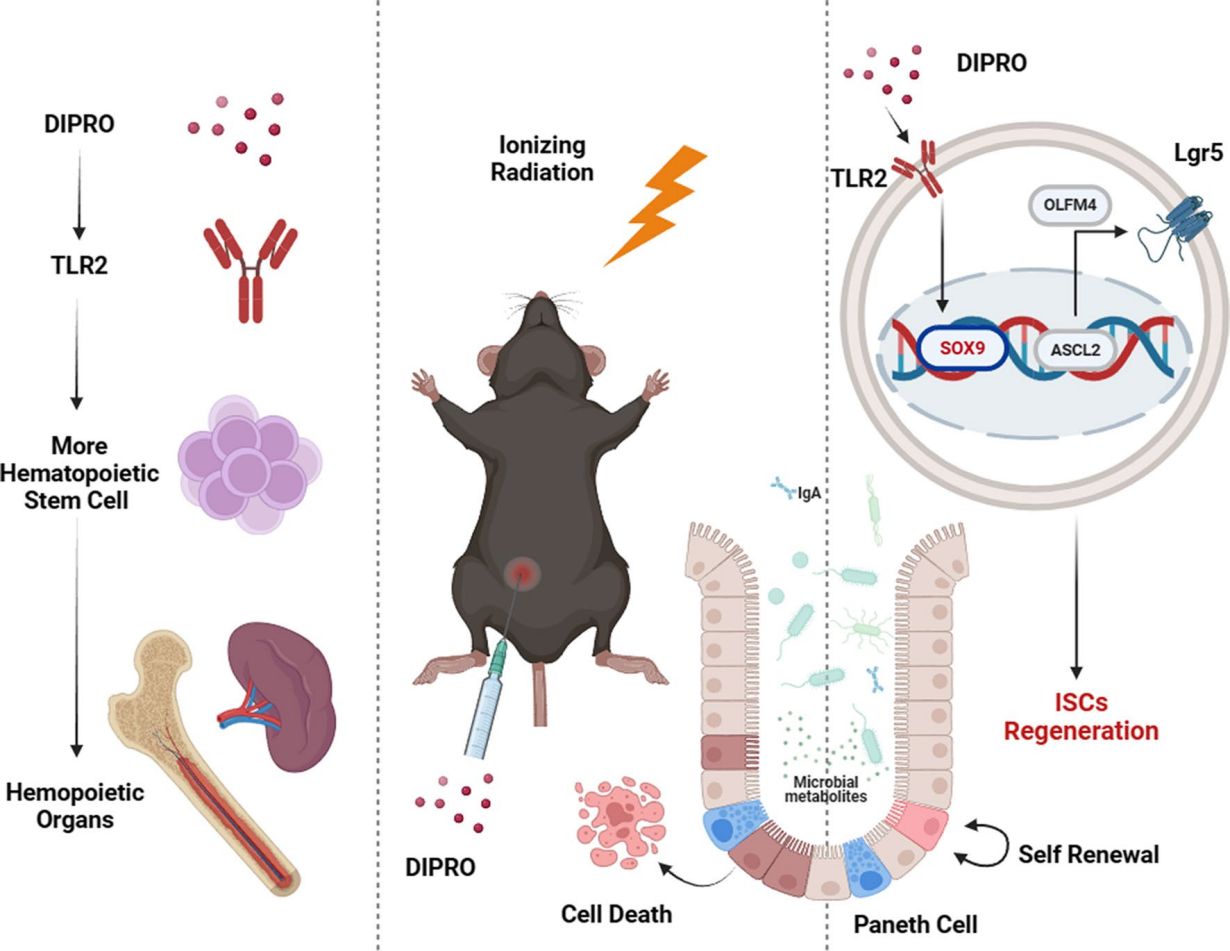

**Supplementary Information:**

The online version contains supplementary material available at 10.1186/s10020-025-01198-2.

## Introduction

With the increasing risk of global nuclear attack and advances in science, technology and medical care, people are at increasing risk of IR exposure, and IR induced injury has become a major public health concern worldwide (Rezvani 2021). High-dose IR exposure can cause life-threatening damage to normal tissues of the human body. The most sensitive to radiation exposure are the hematopoietic and gastrointestinal systems. IR exposure can lead to hematopoietic dysfunction (Dainiak 2018; Williams and McBride 2011; Kiang and Olabisi 2019). This is mainly due to the inability of bone marrow hematopoietic stem and progenitor cells (HSPCs) to maintain normal hematopoiesis after IR. HSCs are the cells of origin of all cell lineages in the blood system within the organism and are regulated by a combination of endogenous and exogenous cellular factors (Mauch et al. 1988; Shao et al. 2014; Wang et al. 2006). HSPCs are more sensitive to radiation damage because they are constantly dividing in vivo, increasing their susceptibility to DNA damage caused by radiation-generated free radicals (Mauch et al. 1995; Li et al. 2022). Similarly, the gut is equally sensitive to radiation exposure (Gandle et al. 2020), and the gut renews its intestinal epithelial cells every 3–4 days (Reynolds et al. 2014; Ludikhuize et al. 2020). IR exposure can lead to intestinal damage and intestinal stem cell death (Meena et al. 2023).

Toll-like Receptors (TLRs) play a crucial role in inducing or stimulating a wide range of immune and non-immune cell functions (Meena et al. 2023; Kawai and Akira 2011). Researches shows that TLRs agonists have protective effects against IR induced damage (Du et al. 2022; Feng et al. 2022; Liu et al. 2020). Dr. Burdelya LG et al. reported that CBLB502, a TLR5 agonist, is effective against radiation damage (Burdelya et al. 2008). Additionally, TLR2/TLR6 ligand attenuates IR-induced hematopoietic injury in mice and nonhuman primates (Brickey et al. 2023). Furthermore, the TLR2 agonist Zymosan-A promotes intestinal stem cell regeneration through upregulation of ASCL2 (Du et al. 2022). These studies remind us that targeting TLRs can prevent and treat radiation-induced injury.

Diprovocim is a small molecule TLR2 agonist that shows significant immunoadjuvant activity in mice and is more potent than the naturally occurring source of TLR2 agonist (Morin et al. 2018; Wang et al. 2018). In this study, we found that Diprovocim activated the TLR2 signaling pathway and up-regulated the expression of SOX9 and promoted ISCs regeneration, attenuated IR-induced hematopoietic and intestinal tissue damage, and improved the survival rate of mice. Therefore, Diprovocim may be a potential efficient radiation protection agent.

## Materials methods

### Chemicals and reagents

Supplementary Table 1 lists reagents and antibodies used in this study. The primers were obtained from Shenggong Biotech (Shanghai, China). Supplementary Table 2 lists the primers used for qRT-PCR analysis.

### Cell culture and treatment

MODE-K cells were obtained from American Type Culture Collection and cultured in RPMI 1640 with 10% FBS at 37 °C in a 5% CO_2_ humidified chamber. After CCK-8 pre-experiments, cells were treated with Diprovocim (0.5 μM) or PBS for 12 h before irradiation.

### CCK-8 assay

Cells were seeded in 96-well plates with 5 × 10^3^ per well. At 24 h after irradiation, cell counting kit-8 reagent was added and the cells were incubated at 37 °C for 2 h. Absorbance was measured in a microplate reader (Beckman Coulter, USA) at 450 nm.

### Apoptosis assay

Apoptosis was analyzed using the apoptosis detection kit. After radiation, Bone marrow cells (BMCs) in mice were isolated, then the cells were stained using Annexin V-fluorescein isothiocyanate (AV-FITC) and propidium iodide-phycoerythrin (PI-PE). The cells were then analyzed by flow cytometry (Beckman Coulter) in accordance with the manufacturer’s instructions.

### Animals and treatment

Male C57BL/6 mice aged 6–8 weeks old were obtained from China Academy of Science (Shanghai, China). Male TLR2 KO mice aged 6–8 weeks old were purchased from Cyagen (Jiangsu, China). All mice were housed in a laboratory animal room under standard conditions. The experiments were approved by the Laboratory Animal Center of the Naval Medical University, China, in conformance with the National Institute of Health Guide for the Care and Use of Laboratory Animals. The number of mice in each group was greater than or equal to 3. After reviewing the literature (Wang et al. 2018), mice were injected intraperitoneally with Diprovocim (10 mg/kg) or saline 18 and 2 h before IR.

### Irradiation

^60^Co (Naval Medical University, China) was used to establish IR induced injury model. The mice were irradiated at 7.5 Gy, 9.5 Gy and 12.0 Gy to establish the Total Body Irradiation (TBI) model. Irradiation of MODE-K cells and the intestinal organoid was performed with the designated dose.

### Cytokine measurement

Serum was collected from mice 3.5 days after IR. The levels of inflammatory cytokines in serum were measured using the LEGENDplex™ Mouse Inflammation Panel kit (cat. 740446, Biolegend, San Diego, CA, USA) according to the manufacturer’s instructions. The beads were analyzed using Flow Cytometer system (Beckman Coulter, USA).

### Flow cytometry

The femurs of mice subjected to the corresponding treatments were collected. Repeatedly rinse the mouse femur 3 times with 1 mL of PBS. The cell suspension was then centrifuged at 1500 rpm for 5 min. After discarding the supernatant, the erythrocytes were lysed with 1 mL of Red Blood Cell Lysis Buffer for 15 min at 4 °C to remove the erythrocytes. The remaining BMCs were washed and resuspended in 1 mL of PBS. After fixation with Binding Buffer, staining was performed and detected by flow cytometry after 20 min.

### Histological examination

Duodenal tissue from the lower part of the stomach of mice was collected and then fixed in 4% paraformaldehyde. Tissue specimens, following fixation, were subjected to dehydration in a graded ethanol series (70%, 85%, and 95% ethanol). Subsequently, the tissues were processed in xylene I and II for clearing. After clearing, the tissues were embedded in molten paraffin. The paraffin-embedded tissue blocks were then cooled and solidified. These blocks were mounted onto a microtome and sectioned into slices with a thickness of 5–8 µm. The sections were dewaxed in an environmentally friendly dewaxing solution, stained with hematoxylin, dehydrated in ethanol, and subsequently stained with eosin. Following dehydration and mounting, the sections were examined under a microscope, and images were captured for analysis.

### Intestine immunofluorescence

Immunofluorescence analysis was used to detect OLFM4 and SOX9 expression. The small intestine was removed and fixed in 4% paraformaldehyde for 25 min and permeabilized in 0.5% Triton X-100 for 10 min. After blocking in BSA, the intestinal tissues were stained with antibodies, followed by the secondary antibody (1:1000). The images were obtained with a fluorescent microscope.

### Intestinal organoid culture

The small intestine was removed from the mice, cut longitudinally and rinsed with cold PBS. The villi were gently scraped off and the remaining tissue was washed about 20 times with cold PBS containing 1% penicillin–streptomycin. The tissue was cut into 2–3 mm pieces, transferred to 15 mM EDTA/PBS and incubated for 2 h at 4 °C. After incubation, the tissue fragments were shaken vigorously and centrifuged three times at 300 rpm/min with cold PBS for 5 min each time. The isolated crypts were then embedded in Matrigel (Corning, New York, USA) and cultured in crypt culture medium (IntestiCult™ Organoid Growth Medium, Stem Cell technologies, Canada).

For radiation experiments, isolated mouse intestinal crypts suspended in Matrigel (250 crypts/50 μL per well) were placed at the center of 24-well plates. Next, 500 μL organoid growth medium was dispensed to each well. Organoids were irradiated 24 h after inoculation. On day 5 after irradiation, mature organoids were observed under a microscope. For radiation response assays, the surface area and budding situation of intestinal organoids were measured by using Image J (National Institutes of Health, Bethesda, MD, USA).

### RNA extraction

Treated cells or tissue fragments were placed in 1 mL of TRIzol reagent and thoroughly mixed. Subsequently, 1/5 volume of chloroform was added, and the mixture was vortexed to ensure uniformity. The sample was then centrifuged at 12,000 rpm for 20 min at 4 °C. The aqueous phase was carefully collected and an equal volume of isopropanol was added to precipitate the RNA. The mixture was centrifuged again at 12,000 rpm for 15 min at 4 °C. The RNA was washed with pre-chilled ethanol, followed by centrifugation to remove the ethanol. Finally, the RNA was dissolved in DEPC-treated water and stored at − 80 °C for further use.

### RNA sequencing and functional enrichment analysis

Total RNA was isolated from the intestine of mice using Trizol (Invitrogen, USA) 24 h after radiation. NanoVue (GE, USA) was used to assess RNA purity. Sequencing was performed at Oebiotech (Shanghai, China) with the Illumina HiSeq 2500 system. Prior to sequencing, the raw data were filtered to produce high-quality clean data. All the subsequent analyses were performed using clean data. All the differentially expressed genes (DEGs) were used for the analysis of heat maps and KEGG ontology enrichment analyses.

### Western blot

Total protein from the MODE-K cells was extracted using mammalian protein extraction reagent. A total of 10^7^ cells were seeded into a 60 mm culture dish. After 12 h, the cells were subjected to the specified treatment. Subsequently, the treated cells were washed three times with PBS and lysed for 15 min in 100 µL of extraction buffer containing a mixture of protease and phosphatase inhibitors. The lysed cells were collected into a 1.5 mL centrifuge tube, sonicated, and then centrifuged at 12,000 rpm for 15 min. The supernatant was collected and then analyzed by western blotting (WB) to detect β-Actin, Myd88, TLR2, p-p38, p-JNK, p-ERK, and SOX9.

### Statistical analysis

Each experiment was independently repeated three times. Representative data are shown. All the values were expressed as means ± the standard errors of means. Two-tailed Student’s t-test was used to analyze the differences between two groups. One-way ANOVA was employed to analyze the differences among three groups. Kaplan–Meier analysis was applied to estimate the difference in overall survival between two groups. The data were analyzed using SPSS ver. 19 software (IBM Corp, Armonk, New York, USA). *p* < 0.05 was considered statistically significant. ns stands for not statistically significant.

## Results

### Diprovocim significantly ameliorated IR induced damage

**Fig. 1:**
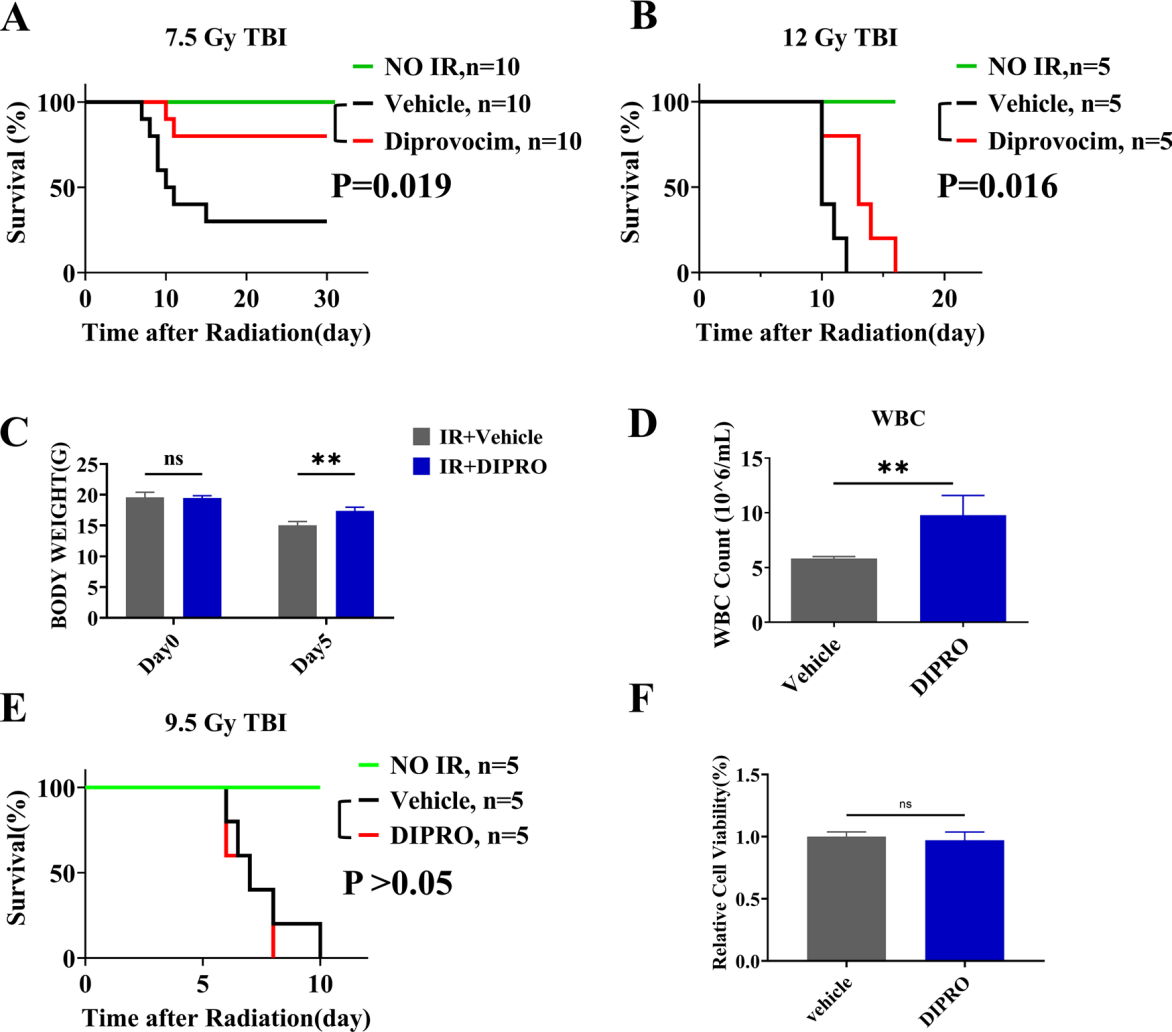
Diprovocim significantly ameliorated IR-induced damage*.* **A, B** Male mice injected intraperitoneally with Diprovocim at 18 h and 2 h before IR, and then exposed to 7.5 Gy, 12.0 Gy TBI. Control mice were treated with Normal Saline. Survival was recorded. **C** The change of body weight from Day 0 to Day 5 in each group of mice after 12 Gy TBI (n = 3). **D** Mice were treated with vehicle or Diprovocim, irradiated at 12 Gy for 1 day, and the count of WBC was determined by using blood cell analyzer (n = 3). **E** Male mice were injected intraperitoneally with Diprovocim at 18 h and 2 h after 9.5 Gy TBI. Control mice were treated with Normal Saline. Survival was recorded. **F** MODE-K cells were stimulated with Diprovocim (0.5 μM) 2 h after 8 Gy IR, and cell viability was determined by CCK-8 (n = 6). The data were presented as mean ± SD. *p < 0.05, **p < 0.01, ns indicates no statistical significance

To determine the radioprotective effects of Diprovocim, C57BL/6 mice were treated with Normal saline (NS) or Diprovocim intraperitoneally 18 h and 2 h before 7.5 Gy or 12 Gy TBI, then the survival of C57BL/6 mice was recorded. As shown in Fig. 1A, B, Diprovocim significantly prolonged the survival of mice after IR exposure. We monitored the body weight of the mice and recorded the changes in body weight of the mice before irradiation and 5 days after 12.0 Gy of IR. As shown in Fig. 1C, Diprovocim alleviated the weight loss of mice after IR. Peripheral blood were extracted from irradiated mice and we found that Diprovocim significantly ameliorated WBC loss in irradiated mice (Fig. 1D). In addition, we found Diprovocim had no therapeutic efficacy on mice and cell after IR (Fig. 1E, F). In sum, these results suggested that Diprovocim significantly ameliorated IR induced damage.

### Diprovocim protected the intestine against IR-induced injury

**Fig. 2:**
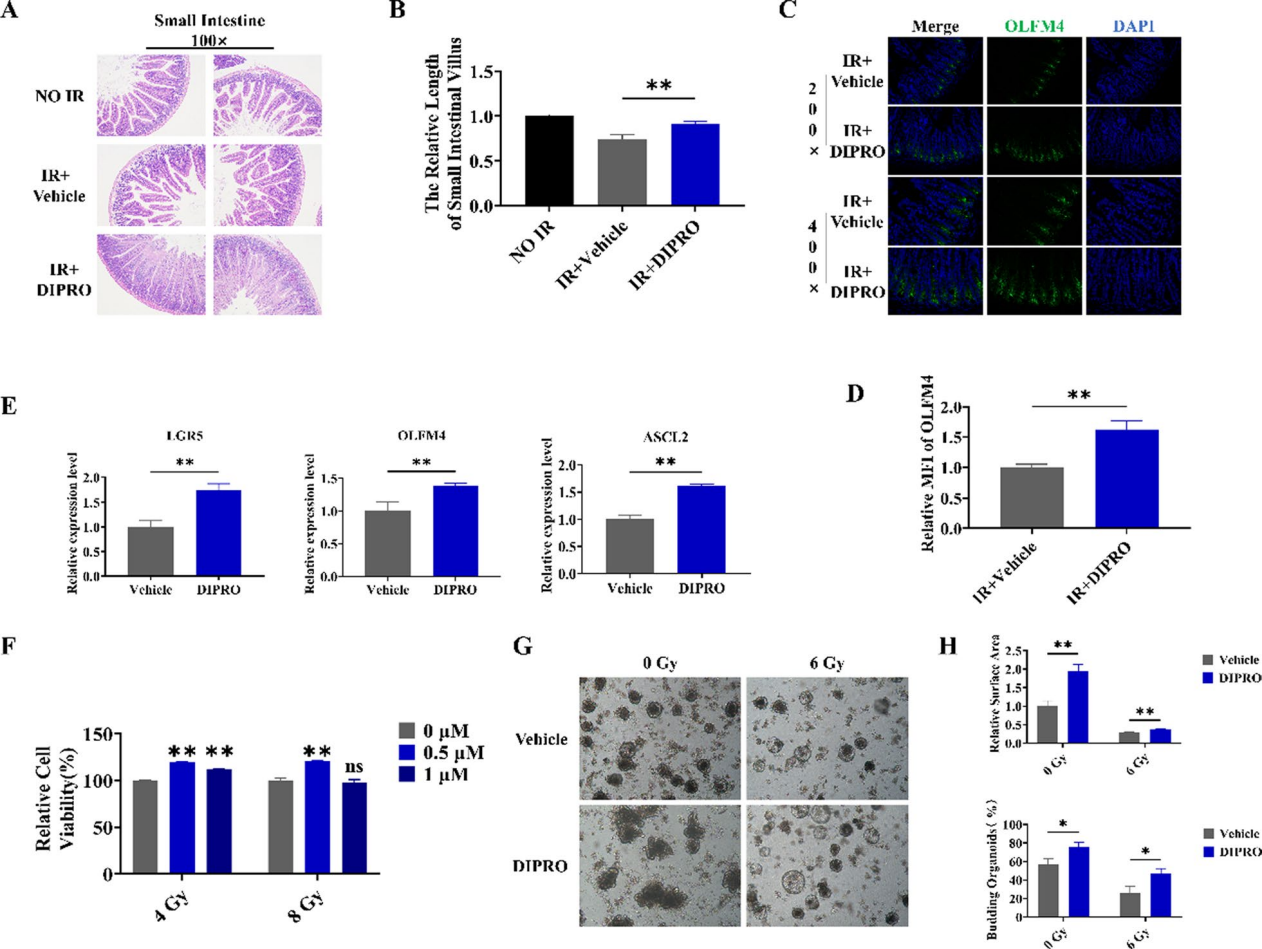
Diprovocim protected the intestinal tissue against radiation-induced injury. **A** Representative images of HE-stained intestinal sections at 3.5 day after 9.5 Gy TBI (n = 3). **B** Villus length (n = 3). **C** The representative images of OLFM4 immunofluorescences intestinal sections at 3.5 days after 9.5 Gy TBI (n = 3). **D** The relative MFI of OLFM4. **E** qRT-PCR results of Lgr5, OLFM4 and ASCL2 (n = 3). **F** The radioprotection of Diprovocim on MODE-K cells viability as determined by CCK-8 assay (n = 6). **G** Intestinal crypts of C57BL/6 mice were extracted for organoid culture, and then it was stimulated with Diprovocim or vehicle. **H** The relative area of intestinal organoids and the percent of budding intestinal organoids. The data were presented as mean ± SD. *p < 0.05, **p < 0.01, ns indicates no statistical significance

HE staining was used to estimate the intestinal radioprotection of Diprovocim. We found Diprovocim significantly ameliorated IR induced intestinal damage in mice, with better intestinal integrity and longer villus lengths in the Diprovocim-administered group (Fig. 2A, B). Intestinal stem cells (ISCs) are the source of various cells in the intestine. The results of OLFM4 immunofluorescence showed that ISCs marker OLFM4 was significantly higher in the Diprovocim group compared with the control group (Fig. 2C, D). Meanwhile, qRT-PCR analysis of intestinal RNA showed that the expression of ISCs markers LGR5, OLFM4, and ASCL2 were increased in the Diprovocim group, which suggesed Diprovocim could promote ISCs regeneration (Fig. 2E). In addition, CCK-8 was conducted and we found Diprovocim also had a significant radioprotection on the viability of MODE-K cells (Fig. 2F). Then we assessed the radioprotective effect of Diprovocim on the intestinal organoids. As shown in Fig. 2G, H, Diprovocim-stimulated organoids exhibited more budding rate and larger surface area both in irradiated and non-irradiated conditions, suggests that Diprovocim can significantly promote the proliferation and differentiation of ISCs after irradiation. Taken together, these results suggested that Diprovocim may attenuate intestinal injury by promoting ISCs regeneration in vivo and in vitro.

### Diprovocim protected the hematopoietic system against radiation-induced injury

**Fig. 3:**
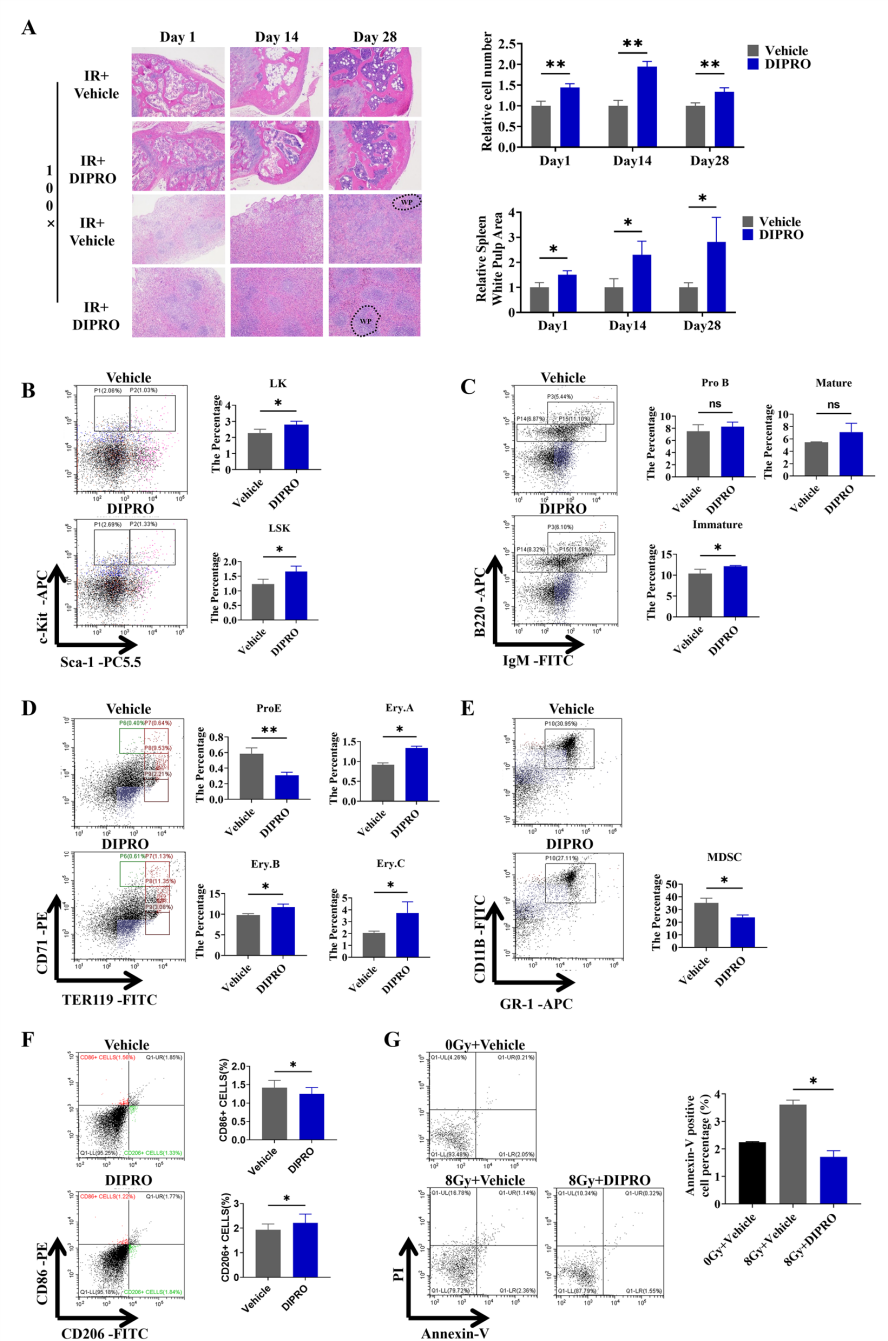
Diprovocim protected the hematopoietic system against radiation-induced injury. **A** Representative images of HE-stained spleen and bone marrow sections after 1 d,14 d, and 28 d of 8.0 Gy IR (n = 3). **B**–**G** Bone marrow cells (BMCs) in mice were isolated at 24 h after 8.0 Gy TBI for flow cytometry analysis (n = 3). **B** The proportions of LK, LSK ratio was detected by using flow cytometry. **C** The proportions of pre-mature B cells, immature B cells and mature B cells was detected by using flow cytometry. **D** The proportion of Pro.E, Ery.A, Ery.B, Ery.C was detected by using flow cytometry. **E** The proportion of MDSC was detected by using flow cytometry. **F** The proportion of macrophage was detected by using flow cytometry. **G** BMCs apoptosis was detected by using flow cytometry (n = 3). The data were presented as mean ± SD. *p < 0.05, **p < 0.01, ns indicates no statistical significance

Next, we discussed whether Diprovocim could protected the hematopoietic system against radiation-induced injury. The HE staining results showed the Diprovocim administration group showed less damage to the spleen and femur than the control group (Fig. 3A). Flow cytometry was performed on the bone marrow cells and the proportions both haematopoietic progenitor cells (lineage^−^scal-1^−^, c-kit^+^, LK) and haematopoietic stem cells (lineage^−^scal-1^+^, c-kit^+^, LSK) were increased in the Diprovocim-administered group (Fig. 3B). Diprovocim also up-regulated the proportion of immature B cells in the bone marrow (Fig. 3C). During erythropoiesis, the proportion of erythroid progenitor cells Pro.E (Erythroid progenitor cells) was decreased in the Diprovocim administration group compared to the control group, whereas the proportions of Ery.A (Basophilic erythroblast), Ery.B (Polychromatophilic erythroblast), and Ery.C (Late erythroblasts) were slao increased (Fig. 3D). While the results of MDSCs suggested Diprovocim down-regulated the proportion of MDSC (CD11b^+^ Gr-1^+^) (Fig. 3E). We also found that Diprovocim promoted M2 (CD86^−^, CD206^+^) polarization in BMDM (Fig. 3F), which reminded us that Diprovocim could suppress the inflammatory response. At the same time, we examined the level of apoptosis of bone marrow cells in mice, and Diprovocim significantly reduced the apoptosis of bone marrow cells caused by IR (Fig. 3G). The above results indicated that Diprovocim protected the hematopoietic system against radiation-induced injury.

### Diprovocim exerted radioprotection through the TLR2 signaling pathway

**Fig. 4:**
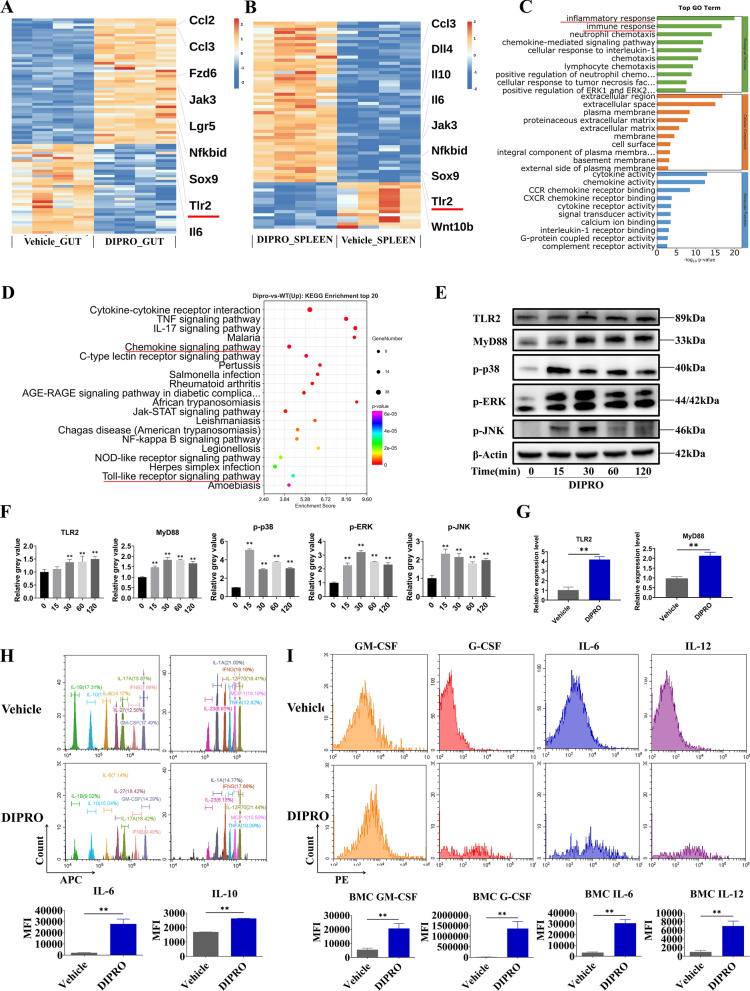
Diprovocim exerted radioprotection through the TLR2 signaling pathway. **A**, **B** Heatmap of DEGs between IR + vehicle mice and IR + Diprovocim mice (n = 4). **C** GO term analysis was performed on DEGs. **D** Pathway enrichment analysis of KEGG. **E** Immunoblot analysis of lysates of MODE-K with 0.5 μM Diprovocim at the indicated times. **F** The quantitative analysis of protein. **G** The expression of TLR2 and MyD88 in intestinal tissue with vehicle or Diprovocim treatment (n = 3). **H** The levels of IL-6 and IL-10 in mouse serum (n = 3). **I** Mean fluorescence intensity of GM-CSF, G-CSF, IL-6 and IL-12 was measured by flow cytometry (n = 3). The data were presented as mean ± SD. *p < 0.05, **p < 0.01

To further explore the radioprotective mechanism of Diprovocim, we performed RNA sequencing (RNA-seq) technique on mouse spleen and intestinal tissues. Differentially expressed genes (DEGs) were screened after secondary analyses (Fig. 4A, B) (Supplementary Table 3, Supplementary Table 4). The GO results showed that Inflammatory response and Immune response were significantly enriched (Fig. 4C) (Supplementary Table 5). Meanwhile, KEGG enrichment analysis showed mmu04620: Toll-like receptor signaling pathway, mmu04062: Chemokine signaling pathway were significantly enriched (Fig. 4D) (Supplementary Table 6). The RNA-Seq results suggested that Diprovocim may exert radioprotective effects by activating the Toll-like receptor signaling pathway. In the list of DEGs, we found that TLR2 was significantly upregulated and Diprovocim has been shown to be a potent ligand for TLR2. It has been shown that TLR2 plays a key role in radioprotection. TLR2 KO mice were more susceptible to radiation-induced morality compared to WT mice. Subsequently, we verified the changes of key proteins in the Toll-like receptor signaling pathway in MODE-K and intestinal tissues. WB results showed that Diprovocim increased the expression of TLR2 and MyD88 and promoted the phosphorylation of p38, JNK and ERK in MODE-K cells. (Fig. 4E, F). In addition, the expression of TLR2 and MyD88 was up-regulated after Diprovocim treatment (Fig. 4G), which was consistent with the previous RNA-Seq results. Meanwhile, we found that IL-6 and IL-10 were up-regulated in serum after Diprovocim treatment (Fig. 4H). The flow cytometry results also showed that the levels of GM-CSF, G-CSF, IL-6 and IL-12 were increased in BMCs after Diprovocim administration (Fig. 4I). Taken together, the above results suggest that Diprovocim may exert radioprotective effects by targeting TLR2.

### The radioprotection of Diprovocim was TLR2 dependent

**Fig. 5:**
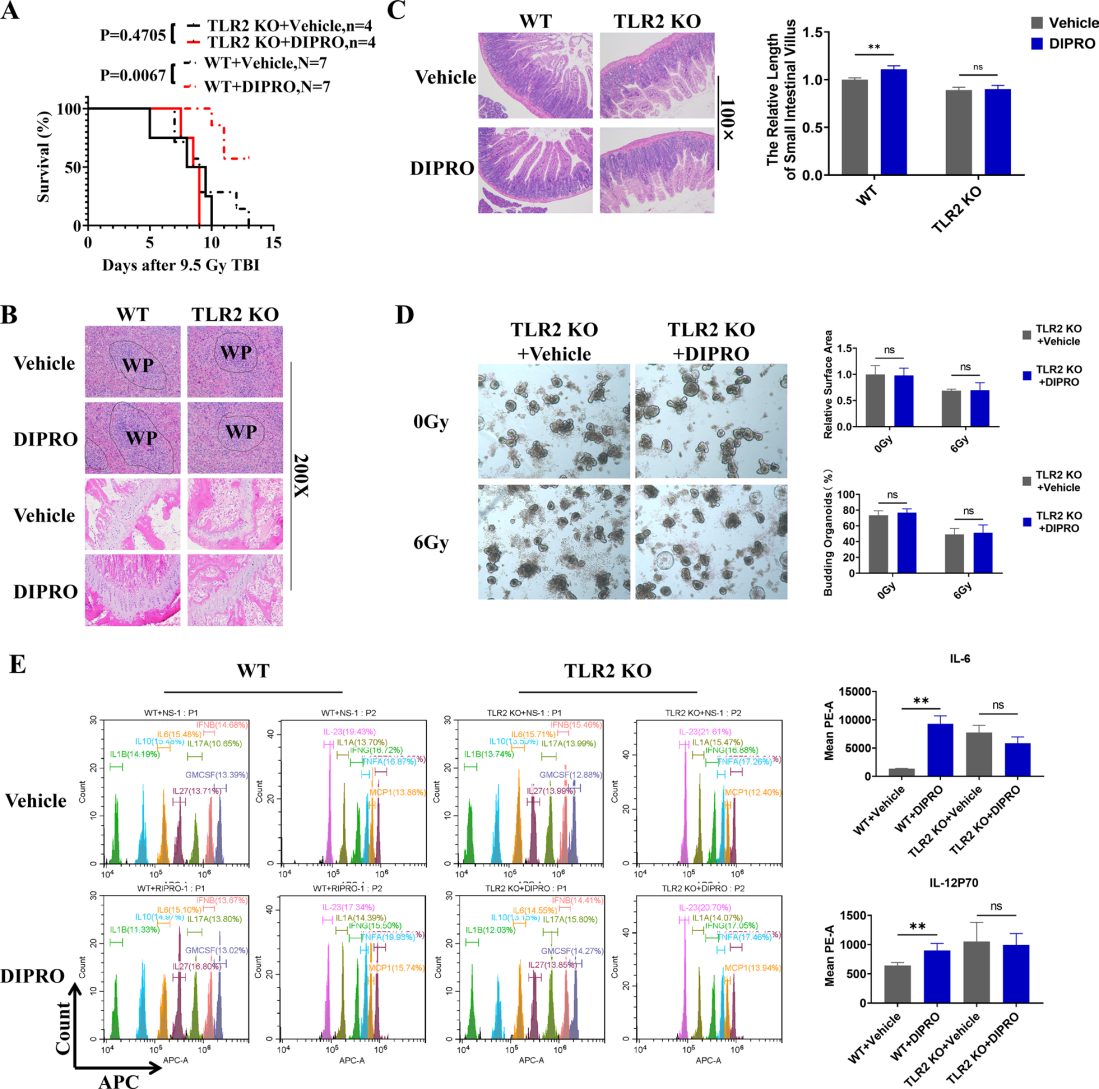
The radioprotection of Diprovocim was TLR2 dependent. **A** C57BL/6 mice and TLR2 KO mice were intraperitoneally injected with vehicle or Diprovocim before 9.5 Gy TBI. Survival was recorded. **B** Representative HE staining images of spleen and bone marrow sections at 3.5 days after 9.5 Gy TBI (n = 3). **C** Representative images of HE-stained intestinal sections after 3.5 days of 9.5 Gy TBI and villus length (n = 3). **D** Intestinal crypts of TLR2 KO mice were extracted for organoid culture, and then it was stimulated with Diprovocim or vehicle. **E** Cytokine changes in serum at day 1 after 9.5 Gy TBI (n = 3). The data were presented as mean ± SD. *p < 0.05, **p < 0.01, ns indicates no statistical significance

The function of TLR2 in Diprovocim-mediated radioprotection were validated by using TLR2 KO mice. The survival results showed that Diprovocim had no radioprotective effects on TLR2 KO mice (*p* = 0.4705) (Fig. 5A). HE staining results of spleen and femur suggested that the radioprotective effect of Diprovocim was significantly diminished in TLR2 KO mice (Fig. 5B). At the same time, pathologic analysis of small intestine proved that Diprovocim had no radioprotective effects on TLR2 KO mice, and there was no significant difference in villus length between Diprovocim and Vehicle group (Fig. 5C). Diprovocim also had no radioprotective effects on intestinal organoids after TLR2 konckout (Fig. 5D). in addition, TLR2 downstream-related factors in mouse serum were also not up-regulated after Diprovocim administration (Fig. 5E). Taken together, these results suggested that the radioprotection of Diprovocim was TLR2 dependent.

### The intestinal radioprotection of Diprovocim was dependent on SOX9

**Fig. 6:**
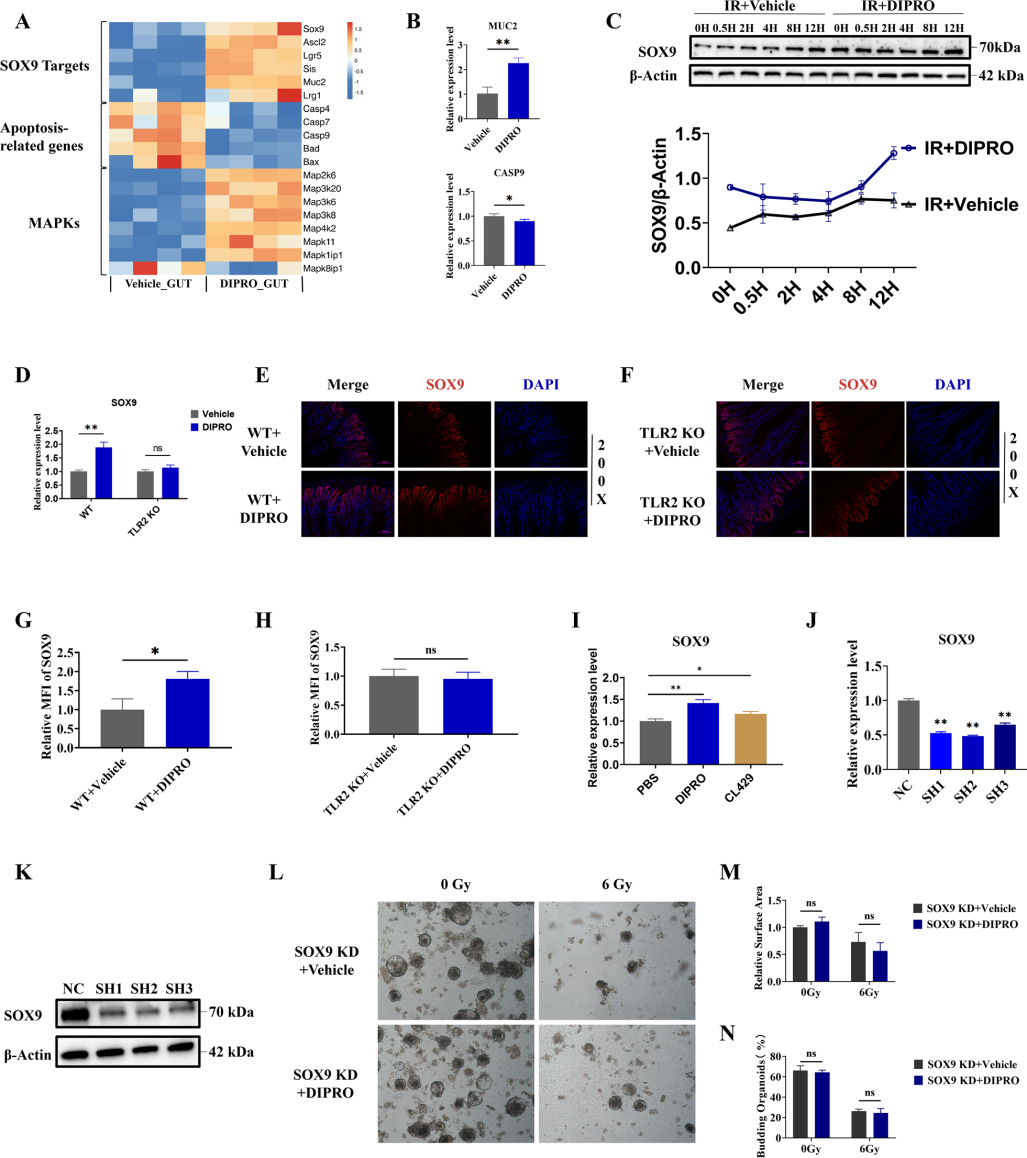
The intestinal radioprotection of Diprovocim was dependent on SOX9. **A** The DEGs of SOX9 downstream target genes, apoptosis-related genes, and MAPK signaling bteween IR + vehicle mice and IR + Diprovocim mice (n = 4). **B** qRT-PCR results of MUC2 and CASP9 in the intestine at 3.5 days after 9.5 Gy TBI (n = 3). **C** WB analysis of MODE-K cells proteins. **D** qRT-PCR results of SOX9 in intestinal tissue of C57BL/6 mice and TLR2 KO mice with vehicle or Diprovocim treatment at 3.5 days after 9.5 Gy TBI (n = 3). **E** The representative images of SOX9 immunofluorescences in WT mice at 3.5 days after 9.5 Gy TBI (n = 3). **F** The representative images of SOX9 immunofluorescences in TLR2 KO mice at 3.5 days after 9.5 Gy TBI (n = 3). **G** The relative MFI of SOX9 in WT mice. **H** The relative MFI of SOX9 in TLR2 KO mice. **I** The expression of SOX9 in MODE-K cells after treatment with the Diprovocim (0.5 μM) or CL429 (2 μg/mL). **J** The relative expression of SOX9 after shRNA knockdown. **K** The validation of SOX9 knockdown at the protein level. **L** Intestinal crypts of C57BL/6 mice were extracted and used to establish SOX9 KD intestinal organoids, and then it was stimulated with Diprovocim or vehicle. **M** Relative surface of intestinal organoids. **N** Budding rate of intestinal organoids. The data were presented as mean ± SD. *p < 0.05, **p < 0.01, ns indicates no statistical significance

RNA-Seq results showed that Diprovocim significantly regulated the expression of genes associated with ISCs, apoptotic response and MAPK pathway (Fig. 6A) (Supplementary Table 7). Among these genes, we found that SOX9 was significantly upregulated by Diprovocim. SOX9 is an important stem cell transcription factor that plays an important role in the development and functional maintenance of various organs and tissues, including cartilage, testis, skin, mammary gland, and intestine (Akiyama et al. 2005; Jakob and Lovell-Badge 2011; Kadaja et al. 2014; Guo et al. 2012; Formeister et al. 2009). MUC2 plays an important role in maintaining intestinal barrier function and homeostasis (Yao et al. 2021). CASP9 is a key initiator of the caspase cascade in the cell apoptotic process (Würstle et al. 2012; Bergstrom et al. 2010) Based on the recent studies and the analysis of RNA-Seq results, we tried to clarify the changes of these DEGs after Diprovocim treatment. The results proved that Diprovocim significantly regulated the expression of MUC2 and CAPS9 (Fig. 6B). Meanwhile, WB results suggested that Diprovocim could up-regulate the expression of SOX9 in MODE-K cells (Fig. 6C). As shown in Fig. 6D, the expression of SOX9 in the intestines of WT mice was increased by Diprovocim treatment, whereas the expression of SOX9 in the intestines of TLR2 KO mice was unchanged. The immunofluorescence result suggested that Diprovocim elevated the fluorescence intensity of SOX9 in the irradiated mouse intestine (Fig. 6E, G). This effect was not observed in TLR2 KO mice (Fig. 6F, H). We further investigated whether Diprovocim could specifically induces SOX9. TLR2 ligands CL429 was used to stimulate MODE-K cells, and we found that CL429 also upregulated SOX9 expression, suggesting that the upregulation of SOX9 by Diprovocim was not specific. Instead, it reminded us that TLR2 ligands may have the potential to upregulate SOX9 expression (Fig. 6I). To determine the critical role of SOX9 in Diprovocim mediated radioprotection, we used shRNA technology to knockdown SOX9. After validation, we selected shRNA2 to establish SOX9 KD intestinal organoids (Fig. 6J, K). The results showed that Diprovocim did not promote the growth of intestinal organs after SOX9 KD under 0 Gy or 6 Gy IR exposure (Fig. 6L–N). Based on the above results, we believed that the radiopotective effects of Diprovocim was dependent on SOX9. In conclusion, these results suggested that the intestinal radioprotection of Diprovocim was dependent on SOX9.

## Discussion

Radiation technology has been widely used in the fields of medicine. How to effectively prevent IR induced damage remains an international scientific problem in the field of radiology that has not been overcome (Rezvani 2021). IR can cause damage to hematopoietic system and gastrointestinal system (Mauch et al. 1995; Hauer-Jensen et al. 2014). It has been shown that the hematopoietic system is affected after exposure and resulted in reduced self-renewal of hematopoietic stem cells and damage to the hematopoietic system (Rodrigues-Moreira et al. 2017). At the same time, the intestine is equally sensitive to IR. The intestinal epithelium is rapidly renewed and this renewal is accomplished by ISCs. The intestinal tract is also maintained by ISCs to maintain homeostasis and regeneration of the damaged intestinal epithelium after injury (Hou et al. 2020). In contrast, after IR exposure, impaired proliferation and apoptosis of ISCs inhibit intestinal epithelial cell renewal (Gong et al. 2016; Kiang et al. 2024).

In this study, we systematically investigated the radioprotection of Diprovocim, a TLR2 agonist, on IR-induced hematopoietic system damage and intestinal damage (Su et al. 2019). We found that Diprovocim significantly prolonged the survival of mice exposed to 7.5 Gy and 12 Gy TBI, and promoted cell proliferation and inhibited apoptosis. IR can cause severe damage to tissues and cells, and the immune system may already be compromised. Diprovocim given after IR exposure may not be effective salvage the damage because the damage has already occurred, the immune system may not be able to respond robustly. IR could reduce the number and function of HPCs, leading to damage to the hematopoietic system (Kiang and Olabisi 2019; Shao et al. 2014; Kalashnikova and Belyavsky 2023). Therefore, we examined the proportion of hematopoietic cell populations. Our results showed that intraperitoneal injection of Diprovocim 18H and 2H before TBI could increase the proportion of LK, LSK, immature B cells, and Ery.A, Ery.B, Ery.C in mouse bone marrow. The percentage of MDSC in mouse bone marrow was decreased after Diprovocim treatment. The above results suggested that Diprovocim can reduce the radiation-induced hematopoietic system damage.

The intestinal epithelium plays an important role as a physical barrier (Peterson and Artis 2014). ISCs are the source of various cells in the intestine, and ISCs can proliferate and differentiate into various types of intestinal cells to cope with the damage after intestinal injury. ISCs maintain intestinal homeostasis through proliferation and self-renewal. IR exposure causes ISCs death and impairs their proliferation and self-renewal, leading to intestinal damage (Saha et al. 2016; Park et al. 2021). In this study, we found Diprovocim treatment reduced the severity of intestinal injury and significantly increased the number of ISCs, suggesting that Diprovocim may alleviate the IR-induced intestinal injury by promoting the regeneration of ISCs. Then we used the intestinal organoid technique to explore the function of Diprovocim on ISCs (Sato and Clevers 2013). The intestinal organoid is a three-dimensional cellular models that contain all types of functional intestinal epithelial cells (Sato et al. 2009). The intestinal organoid is a technique for studying ISC function and regeneration in vitro (Takahashi et al. 2021). The results of intestinal organoid showed that Diprovocim-stimulated organoids exhibited more budding rate and larger surface area both in irradiated and non-irradiated conditions. It was confirmed that Diprovocim significantly promoted the regeneration of ISCs in intestinal organoids after IR.

To further investigate the possible mechanisms of Diprovocim radioprotection, we performed RNA-Seq technology. We found that TLR2 and its associated downstream molecules were significantly upregulated in both spleen and intestinal tissues after Diprovocim treatment. Then we verified the changes of TLR2 signaling pathway after Diprovocim treatment. WB results showed Diprovocim increased the expression of TLR2 and MyD88 and promoted the phosphorylation of p38, JNK and ERK in MODE-K cells. Moreover, the expression of TLR2 and MyD88 was also up-regulated in small intestine after Diprovocim treatment, which was consistent with the RNA-Seq results. In sum, these results reminded us that Diprovocim may play radioprotecive effects via TLR2 signaling pathway.

Recent studies reported that TLR is the critical to radioprotection (Kumar and Kumar 2019; Sanguri and Gupta 2018; Leibowitz et al. 2021). TLR2 is a pattern recognition receptor that is of vital importance in the human immune system and belongs to the TLRs family (Akira et al. 2006). TLRs are important components of the innate immune system, recognizing conserved molecular patterns of microorganisms that activate immune responses (Kawai and Akira 2010). In our previous research, we found that TLR2 plays a key role in radiation resistance in vivo (Du et al. 2022; Fang et al. 2024).TLR2 KO mice were more prone to radiation-induced death (Gao et al. 2015). Moreover, by analyzing the survival and pathology of TLR2 KO mice, we found that the radioprotection of Diprovocim was significantly attenuated after TLR2 konckout, suggesting that Diprovocim exerted radioprotection by activating the TLR2 signaling pathway. As a TLR2 ligand, Diprovocim induced the expression of anti-inflammatory and pro-inflammatory cytokines. Although proinflammatory factors may lead to intestinal inflammatory, it has been reported that proinflammatory factors can exert a radioprotective effects. Brian J Leibowitz et al. reported that IFN-β may be useful in the treatment of radiation-induced acute intestinal injuries and may help to improve intestinal barrier function (Leibowitz et al. 2021). Brett I Bell et al. reported that IL-6 is required for survival of intestinal epithelial cells after injury and that blocking IL-6 signaling exacerbates acute intestinal injury after irradiation (Bell et al. 2019). Meanwhile, some radioprotective agents are also able to upregulate inflammatory factors, such as, Zymosan-A and Lipopolysaccharide (Du et al. 2022; Riehl et al. 2000).The RNA-Seq results showed that Diprovocim significantly regulated the expression of genes associated with ISCs regeneration and apoptosis. We found that SOX9 was significantly upregulated after Diprovocim treatment. SOX9 has been shown to regulate the proliferative capacity of intestinal epithelial cell. SOX9 is predominantly expressed in crypt proliferating cells and Paneth cells (Mori-Akiyama et al. 2007). SOX9 maintains ISCs in an undifferentiated state and maintains ISCs homeostasis. Researchers found that the SOX9 KO mice lacked a reserve cell population, and these mice did not recover after exposure to IR (Roche et al. 2015). It has been reported that TLR4 ligand LPS and IL-4 induce the expression of SOX9 in different types of cells or tissues (Shao et al. 2022; Cai et al. 2024). In this study, our results showed that TLR2 ligands, including Diprovocim and CL429, could upregulate SOX9 expression, which reminded us that the activation of TLR2 may have the potential to upregulate SOX9. We also found that the intestinal radioprotection of Diprovocim was significantly inhibited after SOX9 KD. Taken together, these resulted suggested that the intestinal radioprotection of Diprovocim was dependent on SOX9.

The current research showed that not only TLR2, TLR4, TLR5, and TLR9 all played critical roles in radioprotection. It had been reported that TLR4 ligands LPS, MPLA, TLR5 ligand CBLB502, and TLR9 ligand CPG-ODN could alleviate radiation-induced damage. In this study, we focused on exploring the radioprotection of the small molecule compound Diprovocim, and found Diprovocim could protect WT mice from IR induced death. But Diprovocim had no radioprotection on TLR2 KO mice, which suggested that Diprovocim exerted radioprotection by activating the TLR2 signaling pathway. In addition, RNA-SEQ sequencing also suggests that the NOD signaling pathway and IL-17 signaling pathway were upregulated after DIPRO treatment, and our and other studies have confirmed that activation of these pathways can also alleviate IR-intestinal damage. However, due to limitations in experimental conditions, we only used TLR2 KO mice and SOX9 lentivirus to focus on explore the role of TLR2-SOX9 signaling in DIPRO mediated radioprotection. And further studies are needed to determine whether the TLR2/SOX9 signaling pathway exerts this radiation protection function through other signaling pathways, such as NF-κB/STAT3 or miR-34a/C3/IL-18 (Kiang et al. 2010, 2023). The interaction between these pathways may provide a new perspective to study the regeneration of ISCs after injury.

In comparison with the literature that reviews the radiation MCMs (Horseman et al. 2024), Diprovocim is a small molecule compound with a well-defined target. Diprovocim has been shown to offer significant protection against radiation-induced damage to both the hematopoietic and gastrointestinal systems. This dual protective effect is particularly valuable, as radiation exposure can cause severe damage to these radiation-sensitive systems, leading to both hematopoietic and gastrointestinal acute radiation syndromes.

## Conclusion

In summary, Diprovocim protected the hematopoietic system and intestinal tissues from radiation damage by activating the TLR2 signaling pathway. Diprovocim promoted regeneration of ISCs by upregulating SOX9. Diprovocim may be an effective radioprotective drug for the prevention and treatment of IR-induced intestinal injury.

## Supplementary Information

Additional file 1.

Additional file 2.

Additional file 3.

Additional file 4.

Additional file 5.

Additional file 6.

Additional file 7.

Additional file 8.

## Data Availability

No datasets were generated or analysed during the current study.

## References

[CR1] Akira S, Uematsu S, Takeuchi O. Pathogen recognition and innate immunity. Cell. 2006;124:783–801. 10.1016/j.cell.2006.02.015. 16497588 10.1016/j.cell.2006.02.015

[CR2] Akiyama H, Kim JE, Nakashima K, Balmes G, Iwai N, Deng JM, Zhang Z, Martin JF, Behringer RR, Nakamura T, de Crombrugghe B. Osteo-chondroprogenitor cells are derived from Sox9 expressing precursors. Proc Natl Acad Sci U S A. 2005;102:14665–70. 10.1073/pnas.0504750102. 16203988 10.1073/pnas.0504750102 PMC1239942

[CR3] Bell BI, Koduri S, Salas Salinas C, Monslow J, Puré E, Ben-Josef E, Koumenis C, Verginadis II. Interleukin 6 signaling blockade exacerbates acute and late injury from focal intestinal irradiation. Int J Radiat Oncol Biol Phys. 2019;103:719–27. 10.1016/j.ijrobp.2018.10.007. 30336264 10.1016/j.ijrobp.2018.10.007 PMC7699458

[CR4] Bergstrom KS, Kissoon-Singh V, Gibson DL, Ma C, Montero M, Sham HP, Ryz N, Huang T, Velcich A, Finlay BB, et al. Muc2 protects against lethal infectious colitis by disassociating pathogenic and commensal bacteria from the colonic mucosa. PLoS Pathog. 2010;6:e1000902. 10.1371/journal.ppat.1000902. 20485566 10.1371/journal.ppat.1000902 PMC2869315

[CR5] Brickey WJ, Caudell DL, Macintyre AN, Olson JD, Dai Y, Li S, Dugan GO, Bourland JD, O’Donnell LM, Tooze JA, et al. The TLR2/TLR6 ligand FSL-1 mitigates radiation-induced hematopoietic injury in mice and nonhuman primates. Proc Natl Acad Sci U S A. 2023;120:e2122178120. 10.1073/pnas.2122178120. 38051771 10.1073/pnas.2122178120 PMC10723152

[CR6] Burdelya LG, Krivokrysenko VI, Tallant TC, Strom E, Gleiberman AS, Gupta D, Kurnasov OV, Fort FL, Osterman AL, Didonato JA, et al. An agonist of toll-like receptor 5 has radioprotective activity in mouse and primate models. Science. 2008;320:226–30. 10.1126/science.1154986. 18403709 10.1126/science.1154986 PMC4322935

[CR7] Cai XT, Jia M, Heigl T, Shamir ER, Wong AK, Hall BM, Arlantico A, Hung J, Menon HG, Darmanis S, et al. IL-4-induced SOX9 confers lineage plasticity to aged adult lung stem cells. Cell Rep. 2024;43: 114569. 10.1016/j.celrep.2024.114569. 39088319 10.1016/j.celrep.2024.114569

[CR8] Dainiak N. Medical management of acute radiation syndrome and associated infections in a high-casualty incident. J Radiat Res. 2018;59:ii54–64. 10.1093/jrr/rry004. 29509947 10.1093/jrr/rry004 PMC5941165

[CR9] Du J, Fang L, Zhao J, Yu Y, Feng Z, Wang Y, Cheng Y, Li B, Gao F, Liu C. Zymosan-A promotes the regeneration of intestinal stem cells by upregulating ASCL2. Cell Death Dis. 2022;13:884. 10.1038/s41419-022-05301-x. 36266266 10.1038/s41419-022-05301-x PMC9585075

[CR10] Fang L, Cheng Y, Fang D, Feng Z, Wang Y, Yu Y, Zhao J, Huang D, Zhai X, Liu C, Du J. CL429 enhances the renewal of intestinal stem cells by upregulating TLR2-YAP1. Int Immunopharmacol. 2024;138: 112614. 10.1016/j.intimp.2024.112614. 38972212 10.1016/j.intimp.2024.112614

[CR11] Feng Z, Xu Q, He X, Wang Y, Fang L, Zhao J, Cheng Y, Liu C, Du J, Cai J. FG-4592 protects the intestine from irradiation-induced injury by targeting the TLR4 signaling pathway. Stem Cell Res Ther. 2022;13:271. 10.1186/s13287-022-02945-6. 35729656 10.1186/s13287-022-02945-6 PMC9210818

[CR12] Formeister EJ, Sionas AL, Lorance DK, Barkley CL, Lee GH, Magness ST. Distinct SOX9 levels differentially mark stem/progenitor populations and enteroendocrine cells of the small intestine epithelium. Am J Physiol Gastrointest Liver Physiol. 2009;296:G1108-1118. 10.1152/ajpgi.00004.2009. 19228882 10.1152/ajpgi.00004.2009 PMC2696217

[CR13] Gandle C, Dhingra S, Agarwal S. Radiation-induced enteritis. Clin Gastroenterol Hepatol. 2020;18:A39-a40. 10.1016/j.cgh.2018.11.060. 30529730 10.1016/j.cgh.2018.11.060

[CR14] Gao F, Zhang C, Zhou C, Sun W, Liu X, Zhang P, Han J, Xian L, Bai D, Liu H, et al. A critical role of toll-like receptor 2 (TLR2) and its’ in vivo ligands in radio-resistance. Sci Rep. 2015;5:13004. 10.1038/srep13004. 26268450 10.1038/srep13004 PMC4534783

[CR15] Gong W, Guo M, Han Z, Wang Y, Yang P, Xu C, Wang Q, Du L, Li Q, Zhao H, et al. Mesenchymal stem cells stimulate intestinal stem cells to repair radiation-induced intestinal injury. Cell Death Dis. 2016;7:e2387. 10.1038/cddis.2016.276. 27685631 10.1038/cddis.2016.276 PMC5059875

[CR16] Guo W, Keckesova Z, Donaher JL, Shibue T, Tischler V, Reinhardt F, Itzkovitz S, Noske A, Zürrer-Härdi U, Bell G, et al. Slug and Sox9 cooperatively determine the mammary stem cell state. Cell. 2012;148:1015–28. 10.1016/j.cell.2012.02.008. 22385965 10.1016/j.cell.2012.02.008 PMC3305806

[CR17] Hauer-Jensen M, Denham JW, Andreyev HJ. Radiation enteropathy–pathogenesis, treatment and prevention. Nat Rev Gastroenterol Hepatol. 2014;11:470–9. 10.1038/nrgastro.2014.46. 24686268 10.1038/nrgastro.2014.46 PMC4346191

[CR18] Horseman TS, Frank AM, Cannon G, Zhai M, Olson MG, Lin B, Li X, Hull L, Xiao M, Kiang JG, Burmeister DM. Effects of combined ciprofloxacin and Neulasta therapy on intestinal pathology and gut microbiota after high-dose irradiation in mice. Front Public Health. 2024;12:1365161. 10.3389/fpubh.2024.1365161. 38807988 10.3389/fpubh.2024.1365161 PMC11130442

[CR19] Hou Q, Huang J, Ayansola H, Masatoshi H, Zhang B. Intestinal stem cells and immune cell relationships: potential therapeutic targets for inflammatory bowel diseases. Front Immunol. 2020;11:623691. 10.3389/fimmu.2020.623691. 33584726 10.3389/fimmu.2020.623691 PMC7874163

[CR20] Jakob S, Lovell-Badge R. Sex determination and the control of Sox9 expression in mammals. FEBS J. 2011;278:1002–9. 10.1111/j.1742-4658.2011.08029.x. 21281448 10.1111/j.1742-4658.2011.08029.x

[CR21] Kadaja M, Keyes BE, Lin M, Pasolli HA, Genander M, Polak L, Stokes N, Zheng D, Fuchs E. SOX9: a stem cell transcriptional regulator of secreted niche signaling factors. Genes Dev. 2014;28:328–41. 10.1101/gad.233247.113. 24532713 10.1101/gad.233247.113 PMC3937512

[CR22] Kalashnikova M, Belyavsky A. Hematopoietic System under Physiological Conditions and Following Hematopoietic Reconstitution or Stress. Int J Mol Sci. 2023. 10.3390/ijms24108983. 37240328 10.3390/ijms24108983 PMC10219528

[CR23] Kawai T, Akira S. The role of pattern-recognition receptors in innate immunity: update on Toll-like receptors. Nat Immunol. 2010;11:373–84. 10.1038/ni.1863. 20404851 10.1038/ni.1863

[CR24] Kawai T, Akira S. Toll-like receptors and their crosstalk with other innate receptors in infection and immunity. Immunity. 2011;34:637–50. 10.1016/j.immuni.2011.05.006. 21616434 10.1016/j.immuni.2011.05.006

[CR25] Kiang JG, Olabisi AO. Radiation: a poly-traumatic hit leading to multi-organ injury. Cell Biosci. 2019;9:25. 10.1186/s13578-019-0286-y. 30911370 10.1186/s13578-019-0286-y PMC6417034

[CR26] Kiang JG, Jiao W, Cary LH, Mog SR, Elliott TB, Pellmar TC, Ledney GD. Wound trauma increases radiation-induced mortality by activation of iNOS pathway and elevation of cytokine concentrations and bacterial infection. Radiat Res. 2010;173:319–32. 10.1667/rr1892.1. 20199217 10.1667/RR1892.1 PMC10113926

[CR27] Kiang JG, Cannon G, Olson MG, Zhai M, Woods AK, Xu F, Lin B, Li X, Hull L, Jiang S, Xiao M. Ciprofloxacin and pegylated G-CSF combined therapy mitigates brain hemorrhage and mortality induced by ionizing irradiation. Front Public Health. 2023;11:1268325. 10.3389/fpubh.2023.1268325. 38162617 10.3389/fpubh.2023.1268325 PMC10756649

[CR28] Kiang JG, Cannon G, Singh VK. An overview of radiation countermeasure development in radiation research from 1954 to 2024. Radiat Res. 2024;202:420–31. 10.1667/rade-24-00036.1. 38964743 10.1667/RADE-24-00036.1 PMC11385179

[CR29] Kumar S, Kumar R. Role of acemannan O-acetyl group in murine radioprotection. Carbohydr Polym. 2019;207:460–70. 10.1016/j.carbpol.2018.12.003. 30600029 10.1016/j.carbpol.2018.12.003

[CR30] Leibowitz BJ, Zhao G, Wei L, Ruan H, Epperly M, Chen L, Lu X, Greenberger JS, Zhang L, Yu J. Interferon b drives intestinal regeneration after radiation. Sci Adv. 2021;7:eabi5253. 10.1126/sciadv.abi5253. 34613772 10.1126/sciadv.abi5253 PMC8494436

[CR31] Li N, Chen H, Wang J. DNA damage and repair in the hematopoietic system. Acta Biochim Biophys Sin (Shanghai). 2022;54:847–57. 10.3724/abbs.2022053. 35593466 10.3724/abbs.2022053 PMC9909303

[CR32] Liu Z, Cao K, Liao Z, Chen Y, Lei X, Wei Q, Liu C, Sun X, Yang Y, Cai J, Gao F. Monophosphoryl lipid A alleviated radiation-induced testicular injury through TLR4-dependent exosomes. J Cell Mol Med. 2020;24:3917–30. 10.1111/jcmm.14978. 32135028 10.1111/jcmm.14978 PMC7171420

[CR33] Ludikhuize MC, Meerlo M, Gallego MP, Xanthakis D, Burgaya Julià M, Nguyen NTB, Brombacher EC, Liv N, Maurice MM, Paik JH, et al. Mitochondria define intestinal stem cell differentiation downstream of a FOXO/Notch axis. Cell Metab. 2020;32:889-900.e887. 10.1016/j.cmet.2020.10.005. 33147486 10.1016/j.cmet.2020.10.005

[CR34] Mauch P, Rosenblatt M, Hellman S. Permanent loss in stem cell self renewal capacity following stress to the marrow. Blood. 1988;72:1193–6. 2901866

[CR35] Mauch P, Constine L, Greenberger J, Knospe W, Sullivan J, Liesveld JL, Deeg HJ. Hematopoietic stem cell compartment: acute and late effects of radiation therapy and chemotherapy. Int J Radiat Oncol Biol Phys. 1995;31:1319–39. 10.1016/0360-3016(94)00430-s. 7713791 10.1016/0360-3016(94)00430-S

[CR36] Meena SK, Joriya PR, Yadav SM, Kumar R, Meena P, Patel DD. Modulation of radiation-induced intestinal injury by radioprotective agents: a cellular and molecular perspectives. Rev Environ Health. 2023;38:295–311. 10.1515/reveh-2021-0108. 35438851 10.1515/reveh-2021-0108

[CR37] Mori-Akiyama Y, van den Born M, van Es JH, Hamilton SR, Adams HP, Zhang J, Clevers H, de Crombrugghe B. SOX9 is required for the differentiation of paneth cells in the intestinal epithelium. Gastroenterology. 2007;133:539–46. 10.1053/j.gastro.2007.05.020. 17681175 10.1053/j.gastro.2007.05.020

[CR38] Morin MD, Wang Y, Jones BT, Mifune Y, Su L, Shi H, Moresco EMY, Zhang H, Beutler B, Boger DL. Diprovocims: a new and exceptionally potent class of toll-like receptor agonists. J Am Chem Soc. 2018;140:14440–54. 10.1021/jacs.8b09223. 30272974 10.1021/jacs.8b09223 PMC6209530

[CR39] Park M, Kwon J, Youk H, Shin US, Han YH, Kim Y. Valproic acid protects intestinal organoids against radiation via NOTCH signaling. Cell Biol Int. 2021;45:1523–32. 10.1002/cbin.11591. 33724613 10.1002/cbin.11591

[CR40] Peterson LW, Artis D. Intestinal epithelial cells: regulators of barrier function and immune homeostasis. Nat Rev Immunol. 2014;14:141–53. 10.1038/nri3608. 24566914 10.1038/nri3608

[CR41] Reynolds A, Wharton N, Parris A, Mitchell E, Sobolewski A, Kam C, Bigwood L, El Hadi A, Münsterberg A, Lewis M, et al. Canonical Wnt signals combined with suppressed TGFβ/BMP pathways promote renewal of the native human colonic epithelium. Gut. 2014;63:610–21. 10.1136/gutjnl-2012-304067. 23831735 10.1136/gutjnl-2012-304067 PMC3963552

[CR42] Rezvani M. Therapeutic potential of mesenchymal stromal cells and extracellular vesicles in the treatment of radiation lesions—a review. Cells. 2021. 10.3390/cells10020427. 33670501 10.3390/cells10020427 PMC7922519

[CR43] Riehl T, Cohn S, Tessner T, Schloemann S, Stenson WF. Lipopolysaccharide is radioprotective in the mouse intestine through a prostaglandin-mediated mechanism. Gastroenterology. 2000;118:1106–16. 10.1016/s0016-5085(00)70363-5. 10833485 10.1016/s0016-5085(00)70363-5

[CR44] Roche KC, Gracz AD, Liu XF, Newton V, Akiyama H, Magness ST. SOX9 maintains reserve stem cells and preserves radioresistance in mouse small intestine. Gastroenterology. 2015;149:1553-1563.e1510. 10.1053/j.gastro.2015.07.004. 26170137 10.1053/j.gastro.2015.07.004 PMC4709179

[CR45] Rodrigues-Moreira S, Moreno SG, Ghinatti G, Lewandowski D, Hoffschir F, Ferri F, Gallouet AS, Gay D, Motohashi H, Yamamoto M, et al. Low-dose irradiation promotes persistent oxidative stress and decreases self-renewal in hematopoietic stem cells. Cell Rep. 2017;20:3199–211. 10.1016/j.celrep.2017.09.013. 28954235 10.1016/j.celrep.2017.09.013

[CR46] Saha S, Aranda E, Hayakawa Y, Bhanja P, Atay S, Brodin NP, Li J, Asfaha S, Liu L, Tailor Y, et al. Macrophage-derived extracellular vesicle-packaged WNTs rescue intestinal stem cells and enhance survival after radiation injury. Nat Commun. 2016;7:13096. 10.1038/ncomms13096. 27734833 10.1038/ncomms13096 PMC5065628

[CR47] Sanguri S, Gupta D. Mannan oligosaccharide requires functional ETC and TLR for biological radiation protection to normal cells. BMC Cell Biol. 2018;19:9. 10.1186/s12860-018-0161-4. 29945545 10.1186/s12860-018-0161-4 PMC6020349

[CR48] Sato T, Clevers H. Growing self-organizing mini-guts from a single intestinal stem cell: mechanism and applications. Science. 2013;340:1190–4. 10.1126/science.1234852. 23744940 10.1126/science.1234852

[CR49] Sato T, Vries RG, Snippert HJ, van de Wetering M, Barker N, Stange DE, van Es JH, Abo A, Kujala P, Peters PJ, Clevers H. Single Lgr5 stem cells build crypt-villus structures in vitro without a mesenchymal niche. Nature. 2009;459:262–5. 10.1038/nature07935. 19329995 10.1038/nature07935

[CR50] Shao L, Luo Y, Zhou D. Hematopoietic stem cell injury induced by ionizing radiation. Antioxid Redox Signal. 2014;20:1447–62. 10.1089/ars.2013.5635. 24124731 10.1089/ars.2013.5635 PMC3936513

[CR51] Shao C, Jing Y, Zhao S, Yang X, Hu Y, Meng Y, Huang Y, Ye F, Gao L, Liu W, et al. LPS/Bcl3/YAP1 signaling promotes Sox9(+)HNF4α(+) hepatocyte-mediated liver regeneration after hepatectomy. Cell Death Dis. 2022;13:277. 10.1038/s41419-022-04715-x. 35351855 10.1038/s41419-022-04715-x PMC8964805

[CR52] Su L, Wang Y, Wang J, Mifune Y, Morin MD, Jones BT, Moresco EMY, Boger DL, Beutler B, Zhang H. Structural basis of TLR2/TLR1 activation by the synthetic agonist diprovocim. J Med Chem. 2019;62:2938–49. 10.1021/acs.jmedchem.8b01583. 30829478 10.1021/acs.jmedchem.8b01583 PMC6537610

[CR53] Takahashi T, Fujishima K, Kengaku M. Modeling Intestinal Stem Cell Function with Organoids. Int J Mol Sci. 2021. 10.3390/ijms222010912. 34681571 10.3390/ijms222010912 PMC8535974

[CR54] Wang Y, Schulte BA, LaRue AC, Ogawa M, Zhou D. Total body irradiation selectively induces murine hematopoietic stem cell senescence. Blood. 2006;107:358–66. 10.1182/blood-2005-04-1418. 16150936 10.1182/blood-2005-04-1418 PMC1895367

[CR55] Wang Y, Su L, Morin MD, Jones BT, Mifune Y, Shi H, Wang KW, Zhan X, Liu A, Wang J, et al. Adjuvant effect of the novel TLR1/TLR2 agonist Diprovocim synergizes with anti-PD-L1 to eliminate melanoma in mice. Proc Natl Acad Sci U S A. 2018;115:E8698-e8706. 10.1073/pnas.1809232115. 30150374 10.1073/pnas.1809232115 PMC6140543

[CR56] Williams JP, McBride WH. After the bomb drops: a new look at radiation-induced multiple organ dysfunction syndrome (MODS). Int J Radiat Biol. 2011;87:851–68. 10.3109/09553002.2011.560996. 21417595 10.3109/09553002.2011.560996 PMC3314299

[CR57] Würstle ML, Laussmann MA, Rehm M. The central role of initiator caspase-9 in apoptosis signal transduction and the regulation of its activation and activity on the apoptosome. Exp Cell Res. 2012;318:1213–20. 10.1016/j.yexcr.2012.02.013. 22406265 10.1016/j.yexcr.2012.02.013

[CR58] Yao D, Dai W, Dong M, Dai C, Wu S. MUC2 and related bacterial factors: therapeutic targets for ulcerative colitis. EBioMedicine. 2021;74:103751. 10.1016/j.ebiom.2021.103751. 34902790 10.1016/j.ebiom.2021.103751 PMC8671112

