# Supplementary figures and images for: Diprovocim protects against the radiation-induced damage via the TLR2 signaling pathway
Source: Mol Med. 2025 Apr 17;31:139. doi: 10.1186/s10020-025-01198-2 (PMC12004591; doi:10.1186/s10020-025-01198-2)

E

FIGURE 4E

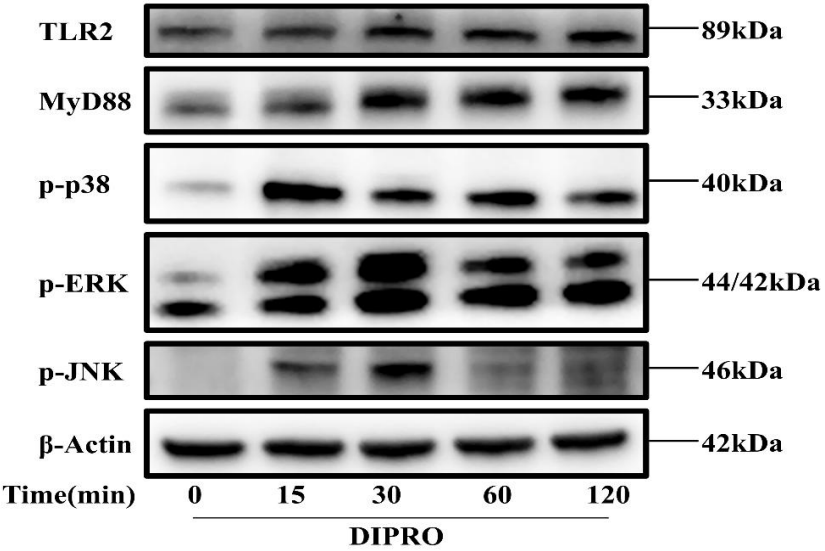

TLR2

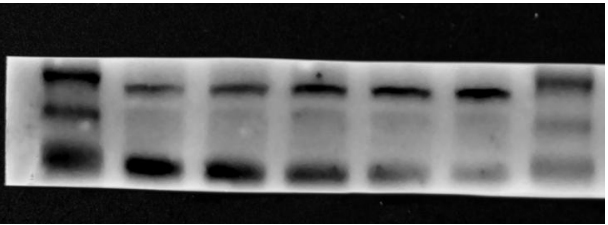

MyD88

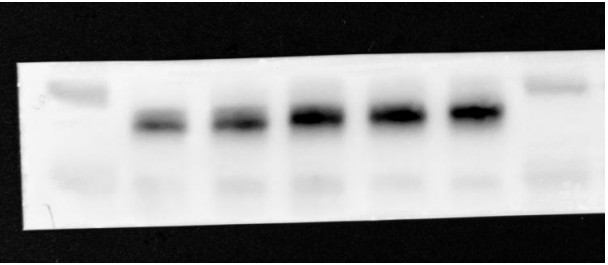

p-p38

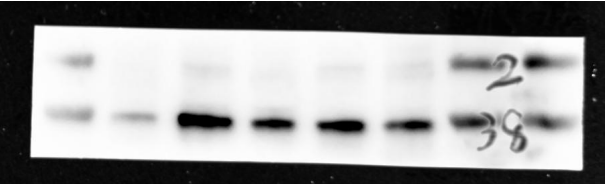

p-ERK

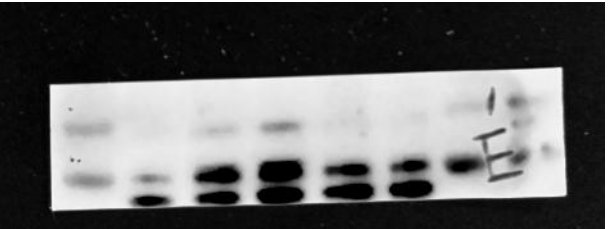

p-JNK

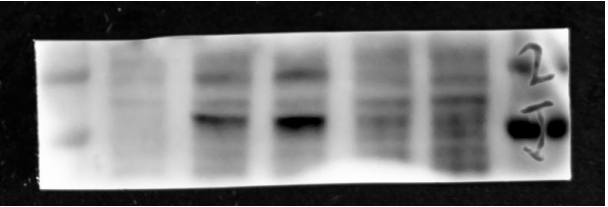

β-Actin

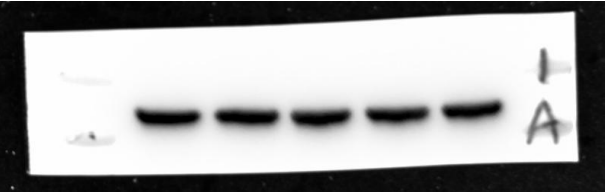

FIGURE 6C

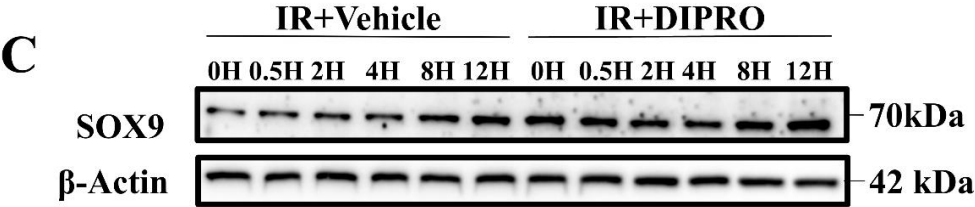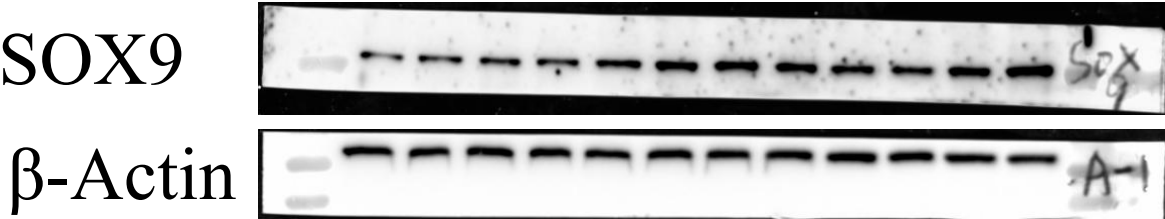

FIGURE 6K

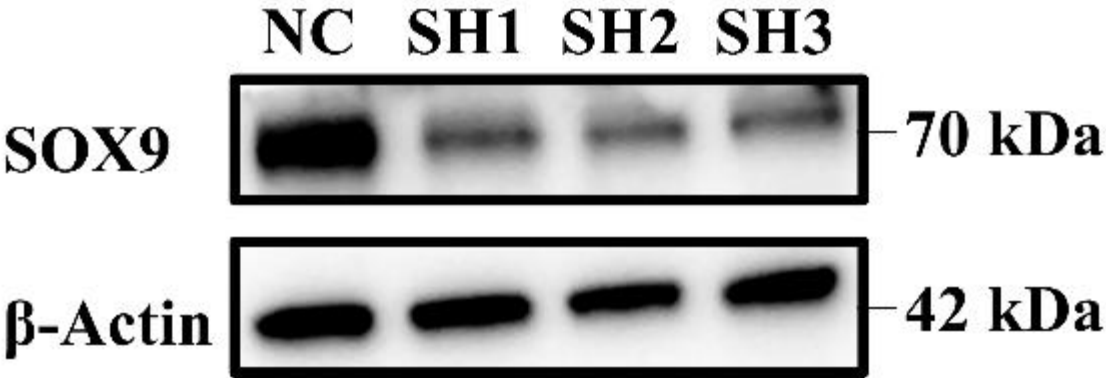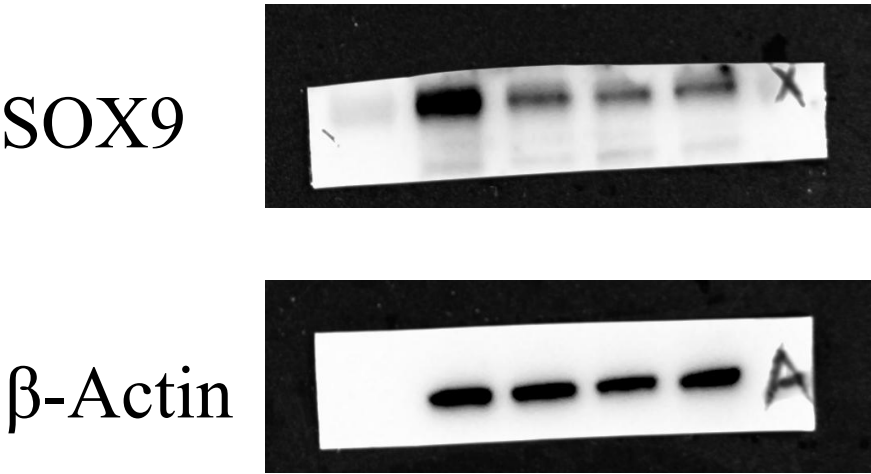

Supplement: Supplementary file 8 — Additional file 8. [file 10020_2025_1198_MOESM8_ESM.pdf]
